# Supplementary material for: The effect of religiosity on violence: Results from a Brazilian population-based representative survey of 4,607 individuals
Source: PLoS One. 2020 Aug 25;15(8):e0238020. doi: 10.1371/journal.pone.0238020 (PMC7447020; doi:10.1371/journal.pone.0238020)

PLOS ONE

The effect of religiosity on violence: results from a nationwide population-based representative survey of 4,607 individuals.

--Manuscript Draft--

**Manuscript Number:**	PONE-D-20-08721
**Article Type:**	Research Article
**Full Title:**	The effect of religiosity on violence: results from a nationwide population-based representative survey of 4,607 individuals.
**Short Title:**	The effect of religiosity on violence: results from a nationwide representative population.
**Corresponding Author:**	Juliane Piasseschi de Bernardin Gonçalves, Ph.D.<br>Universidade de Sao Paulo<br>Sao Paulo, SP BRAZIL
**Keywords:**	violence; police detention; domestic violence; fight; alcohol dependence; religion; spirituality.
**Abstract:**	Although there is a wide array of evidence showing the beneficial effect of religiosity on violence among adolescents, nationwide studies in the general population are scarce. This study aims to explore whether religiosity is associated or not with diminishing violence in a Brazilian population-based representative sample. This observational cross-sectional study was conducted in 2011-2012 using face-to-face interviews and included 4,608 individuals 14 years and older. The survey included measures of religiosity (religious affiliation and importance of religion), violence (involvement in fights, domestic violence and police detention), depression, social support and alcohol dependence. Were used logistic regression models and mediation analyses. In the total sample analyses, after adjustments, religious affiliation was inversely associated with lower involvement in fights (OR=0.60,CI95%:0.37-0.98) and less police detention (OR=0.37,CI95%:0.20-0.70), whereas the importance of religion was only associated with less fights (OR=0.60,CI95%:0.36-0.99). Subanalyses revealed different associations depending on the age group evaluated. Mediation tests showed that the association of religious affiliation on violence outcomes was mediated by alcohol use. In conclusion, religiosity seems to be an important factor associated with lower levels of violence in this nationwide representative survey and alcohol dependence seems to mediate this relationship. Health professionals should be aware of these findings in their clinical practice.
**Order of Authors:**	Juliane Piasseschi de Bernardin Gonçalves, MsC
	Clarice Madruga
	Giancarlo Lucchetti
	Maria do Rosário Latorre
	Ronaldo Laranjeira
	Homero Vallada
**Additional Information:**	
**Question**	**Response**
**Financial Disclosure**<br><br>Enter a financial disclosure statement that describes the sources of funding for the work included in this submission. Review the submission guidelines for detailed requirements. View published research articles from *PLOS ONE* for specific	This work received financial support from CNPq-Brazilian Council for Scientific and Technological Development (GL, HV and RL) and from CAPES-Brazilian Federal Government Agency (JPBG).

Powered by Editorial Manager® and ProduXion Manager® from Aries Systems Corporation

examples.<br><br>This statement is required for submission and **will appear in the published article** if the submission is accepted. Please make sure it is accurate.<br><br>**Unfunded studies**<br>Enter: *The author(s) received no specific funding for this work.*<br><br>**Funded studies**<br>Enter a statement with the following details:<br>• Initials of the authors who received each award<br>• Grant numbers awarded to each author<br>• The full name of each funder<br>• URL of each funder website<br>• Did the sponsors or funders play any role in the study design, data collection and analysis, decision to publish, or preparation of the manuscript?<br>• **NO** - Include this sentence at the end of your statement: *The funders had no role in study design, data collection and analysis, decision to publish, or preparation of the manuscript.*<br>• **YES** - Specify the role(s) played.<br><br>* typeset	
**Competing Interests**<br><br>Use the instructions below to enter a competing interest statement for this submission. On behalf of all authors, disclose any competing interests that could be perceived to bias this work—acknowledging all financial support and any other relevant financial or non-financial competing interests.<br><br>This statement **will appear in the published article** if the submission is accepted. Please make sure it is accurate. View published research articles from *PLOS ONE* for specific examples.	The authors have declared that no competing interests exist.

Powered by Editorial Manager® and ProduXion Manager® from Aries Systems Corporation

**NO authors have competing interests**<br><br>Enter: *The authors have declared that no competing interests exist.*<br><br>**Authors with competing interests**<br><br>Enter competing interest details beginning with this statement:<br><br>*I have read the journal's policy and the authors of this manuscript have the following competing interests: [insert competing interests here]*<br><br>* typeset	
**Ethics Statement**<br><br>Enter an ethics statement for this submission. This statement is required if the study involved:<br><br>• Human participants<br>• Human specimens or tissue<br>• Vertebrate animals or cephalopods<br>• Vertebrate embryos or tissues<br>• Field research<br><br>Write "N/A" if the submission does not require an ethics statement.<br><br>General guidance is provided below. Consult the submission guidelines for detailed instructions. **Make sure that all information entered here is included in the Methods section of the manuscript.**	CONEP 10-043802

**Format for specific study types**<br><br>**Human Subject Research (involving human participants and/or tissue)**<br>• Give the name of the institutional review board or ethics committee that approved the study<br>• Include the approval number and/or a statement indicating approval of this research<br>• Indicate the form of consent obtained (written/oral) or the reason that consent was not obtained (e.g. the data were analyzed anonymously)<br><br>**Animal Research (involving vertebrate animals, embryos or tissues)**<br>• Provide the name of the Institutional Animal Care and Use Committee (IACUC) or other relevant ethics board that reviewed the study protocol, and indicate whether they approved this research or granted a formal waiver of ethical approval<br>• Include an approval number if one was obtained<br>• If the study involved *non-human primates*, add *additional details* about animal welfare and steps taken to ameliorate suffering<br>• If anesthesia, euthanasia, or any kind of animal sacrifice is part of the study, include briefly which substances and/or methods were applied<br><br>**Field Research**<br><br>Include the following details if this study involves the collection of plant, animal, or other materials from a natural setting:<br>• Field permit number<br>• Name of the institution or relevant body that granted permission	
**Data Availability**<br><br>Authors are required to make all data underlying the findings described fully available, without restriction, and from the time of publication. PLOS allows rare exceptions to address legal and ethical concerns. See the PLOS Data Policy and FAQ for detailed information.	Yes - all data are fully available without restriction

Powered by Editorial Manager® and ProduXion Manager® from Aries Systems Corporation

A Data Availability Statement describing where the data can be found is required at submission. Your answers to this question constitute the Data Availability Statement and **will be published in the article**, if accepted.<br><br>**Important:** Stating 'data available on request from the author' is not sufficient. If your data are only available upon request, select 'No' for the first question and explain your exceptional situation in the text box.<br><br>Do the authors confirm that all data underlying the findings described in their manuscript are fully available without restriction?	
**Describe where the data may be found in full sentences. If you are copying our sample text, replace any instances of XXX with the appropriate details.**<br><br>• If the data are **held or will be held in a public repository**, include URLs, accession numbers or DOIs. If this information will only be available after acceptance, indicate this by ticking the box below. For example: *All XXX files are available from the XXX database (accession number(s) XXX, XXX.*).<br>• If the data are all contained **within the manuscript and/or Supporting Information files**, enter the following: *All relevant data are within the manuscript and its Supporting Information files.*<br>• If neither of these applies but you are able to provide **details of access elsewhere**, with or without limitations, please do so. For example:<br><br>*Data cannot be shared publicly because of [XXX]. Data are available from the XXX Institutional Data Access / Ethics Committee (contact via XXX) for researchers who meet the criteria for access to confidential data.*<br><br>*The data underlying the results presented in the study are available from (include the name of the third party*	Data cannot be shared publicly because of confidencial information. Data are available from the Federal University of Sao Paulo Institution

and contact information or URL).<br>• This text is appropriate if the data are owned by a third party and authors do not have permission to share the data.<br><br>* typeset	
Additional data availability information:	

Powered by Editorial Manager® and ProduXion Manager® from Aries Systems Corporation

Manuscript

Click here to download Manuscript Religiosity on violence in a nationwide population.doc

The effect of religiosity on violence: results from a nationwide population-based representative survey of 4,607 individuals.

Authors: Juliane Piasseschi de Bernardin Gonçalves[1], Clarice Sandi Madruga[2], Giancarlo Lucchetti[3], Maria do Rosário Dias Latorre[4], Ronaldo Laranjeira[2], Homero Vallada[1,5]

[1] Department of Psychiatry, Faculdade de Medicina da Universidade de Sao Paulo (LIM-23/ProSER), Sao Paulo, Brazil

[2] Psychiatry department, Federal University of Sao Paulo, Sao Paulo, Brazil

[3] Department of Medicine, School of Medicine, Federal University of Juiz de Fora, Brazil

[4] Department of Epidemiology, School of Public Health, University of Sao Paulo, Sao Paulo, Brazil

[5] Department of Psychiatry, Faculdade de Medicina da Universidade de Sao Paulo (LIM-23/ProSER), Sao Paulo, Brazil

Corresponding author:

Email: juliane.pbg@usp.br (JPBG)

Abstract

Although there is a wide array of evidence showing the beneficial effect of religiosity on violence among adolescents, nationwide studies in the general population are scarce. This study aims to explore whether religiosity is associated or not with diminishing violence in a Brazilian population-based representative sample. This observational cross-sectional study was conducted in 2011-2012 using face-to-face interviews and included 4,608 individuals 14 years and older. The survey included measures of religiosity (religious affiliation and importance of religion), violence (involvement in fights, domestic violence and police detention), depression, social support and alcohol dependence. Were used logistic regression models and mediation analyses. In the total sample analyses, after adjustments, religious affiliation was inversely associated with lower involvement in fights (OR=0.60,CI95%:0.37-0.98) and less police detention (OR=0.37,CI95%:0.20-0.70), whereas the importance of religion was only associated with less fights (OR=0.60,CI95%:0.36-0.99). Subanalyses revealed different associations depending on the age group evaluated. Mediation tests showed that the association of religious affiliation on violence outcomes was mediated by alcohol use. In conclusion, religiosity seems to be an important factor associated with lower levels of violence in this nationwide representative survey and alcohol dependence seems to mediate this relationship. Health professionals should be aware of these findings in their clinical practice.

Keywords: violence, police detention, domestic violence, fight, alcohol dependence, religion, spirituality.

Introduction

The World Health Organization estimates that more than 1.3 million deaths occur every year as the result of violence, which is the fourth leading cause of death worldwide for individuals aged 15-44 years old [1]. A worldwide survey of 187 countries showed a 45% increase in firearm attacks and 32.6% increase in the use of sharp objects from 1990 to 2013 [2]. In addition, violence has a long-term impact on psychological and social outcomes, with economic consequences that include the high costs of treatment, the overuse of mental health services, emergency care and criminal justice [1].

Due to the high prevalence rates of violence, a growing number of preventive initiatives have been developed and implemented [3], which is supported by the latest version of the "Diagnostic and Statistical Manual of Mental Disorders" (DSM-V – 2013) of the American Psychiatric Association that suggests the inclusion of cultural perspectives to the treatment of victims of violence [4]. Among the complementary strategies proposed for mental health rehabilitation programs, there is evidence that interventions based on religiosity/spirituality (R/S) can be beneficial [5]. Likewise, studies are showing the promising role of R/S in diminishing and/or preventing violent behavior [6–8].

The impact of R/S on violence has been noted in the 60s by Hirschi and Stark [9], who hypothesized that religion could prevent individual criminal behavior through supernatural boundaries and rewarding Divine promises. However, further attempts in the 80s failed to confirm this hypothesis [10,11]. More recently, however, representative samples of American adolescents and young adults showed an inverse relationship between levels of R/S and delinquency [12–16].

Moreover, three systematic reviews including rigorous methodological designs showed that R/S may be inversely associated with several risk behavior outcomes in adolescents [6–8]. Despite this evidence, most authors consider involvement with drugs as a “delinquency behavior,” since the use of those substances is prohibited. Therefore, Kelly et al. [8] performed a meta-analysis separating drug delinquency from nondrug delinquency, showing that the magnitude of R/S was different among the measures, as it was stronger for drug use, followed by alcohol use and then by delinquency. The separation into two groups (drug and nondrug delinquency) is a potential approach to further understand this relationship, since R/S has been constantly described as protective for substance use and abuse [5,17].

Despite the growing evidence of this relationship, mediators were also not fully explored by previous authors. Heavy episodic drinking was recently described as a partial mediator of church attendance on sexual aggression perpetration in a sample of male adolescents [18]. However, other drugs and social support, were not investigated as potential mediators of this relationship.

Within this context, this study aims to advance in this field of knowledge, investigating the general population and exploring possible mediators, which were seldom studied in other samples. In 2000, Benda and Toombs [19] reported a moderator effect of age, where individuals with high levels of R/S above 24 years old were less inclined to perpetrate violence. Therefore, the present study aimed to investigate the relationship between R/S and involvement in fights, police detention and domestic violence in a Brazilian large nationally representative sample of general population.

Methods

Study design

This study was part of the Second Brazilian National Alcohol and Drugs Survey (II BNADS), a cross-sectional multistage cluster sampling of alcohol consumption patterns [20]. The protocol was approved by the Ethics Committee of the Federal University of Sao Paulo and by the Brazilian National Commission of Ethics in Research (CONEP 10-043802), Brazil. All subjects provided written informed consent prior to being interviewed.

Setting, participants and eligibility criteria

This Brazilian representative population-based survey was conducted between November 2011 to March 2012 during a face-to-face interview in the respondents' homes with individuals who were at least 14 years old. The process involved three steps: 1) selection of 149 counties using probability proportional to size methods (PPS); 2) selection of 375 census sectors (two for each county), also using PPS; and 3) selection of eight simple random households within each census sector, followed by sample selection of a household member by the "closest future birthday" technique. Individuals with severe cognitive deficiency, those who were absent after three visits and who did not agree to participate as well as those from native Brazilian tribes were not included.

Interview

The one-hour interview was carried out by trained research assistants and was an adaptation of the "Hispanic Americans Baseline Alcohol Survey (HABLAS)" [21], fully checked for

its consistency. In order to guarantee the quality of the data collection, 20% of the interviews were followed by the principal researchers.

Variables used in this study

Dependent Variables

Three different dependent variables of violence were analyzed:

- Involvement in fights: assessed through the question: “In the last twelve months, how often where you involved in a fight with physical aggression?” Answers were dichotomized as “never” (0) or “at least once” (1).
- Domestic violence: only asked for people married/living with a partner: “In the last twelve months, did you do any of these things to your partner: throw things, push, shake, slap, bite, kick, burn, force to have sex or strike with a knife/weapon?”. Answers were dichotomized as “never” (0) or “at least once” (1). Domestic violence represented 56% of the total sample including people between 31-59 years of age.
- Police detention: assessed through the question “In the last twelve months, have you been detained or arrested by the police?”. It could be answered by “yes” (1) or “no (0).”

Independent Variables

Religiosity was assessed in the following way:

- Religious affiliation: appraised using the question “What is your religion?” with the most prevalent religions as possible answers, which were categorized as “none” (0) or “had any” (1).

- Importance of religion: evaluated using the question “How important is the religion in your life?”. Answers were categorized as “not important/indifferent/without religion” (0) and “very important/important” (1).

Other measures were:

- Depression: through the validated Brazilian version of the 20-item Center for Epidemiological Studies Depression Scale (CES-D) [22]. The score ranged from 0 (no depression) to 60 and was classified as “yes” or “no” by the cutoff of 16 points.
- Social support: using an adaptation of the Adult Psychiatric Morbidity Survey of England and categorized results as “high” or “low” using 17 points as the cutoff [23].
- Alcohol dependence: was diagnosed using the DSM-V criteria and classified as “yes” or “no” according to the criteria of the CIDI cutoff [24].
- Sociodemographic variables: age (which was subdivided in four age group: 14-18, 19-30, 31-59, 60 and above), gender (female and male), years of school, Brazilian regions (North, Northeast, Midwest, Southeast, South), marital status (married/living with a partner, single/divorced/widower) and race (classified as: White, Black, Brown and others). Race in Brazil was assessed by the self-report of the color of skin with “Brown” referring to a mix of different races (predominantly refers to European and African ancestries but could also be related to Amerindians and African ancestries).

Statistical methods

STATA 13.0 was used for all analyses which were weighted due to the stratified sampling design and non-responses using post-stratification. All individuals who refused or answered “I

do not know" in any of the questions were excluded, and they represented 0.2% of the total sample. First, a descriptive analysis was carried out using absolute number and percentages, means and standard deviations for each dependent and independent variable.

To analyze the relationship between violence and religiosity, we used chi-square tests and logistic regression models (OR-*odds ratio*) with 95% confidence intervals. Models were reported in an unadjusted and in an adjusted way for sociodemographic variables (age, gender, education, marital status), depression, social support and alcohol dependence. These models were verified in the general population and also in age group sub analysis.

Finally, in order to verify the possible mediator role of different independent variables in the relationship between religious variables and violence outcomes, we carried out a series of mediation models [25]. To explore this mediation models, Chi-square tests (CI:95%) were used to verify if the variables met the following criteria: a) religiosity was associated with mediators; b) religiosity was associated with violence without mediators; c) mediators had a significant effect on violence; and d) the effect of religiosity on violence was attenuated after addition of the mediator [26]. If the criteria were met, Sobel-Goodman tests [27] were used to identify if there was a partial or total mediation effect. Partial mediation is showed when there are significant levels on both direct and indirect pathways and total mediation means that there is only significance on indirect effect [28]. A $p<0.05$ was considered significant in all analyses.

Results

The dataset was composed of 4,607 individuals, representing 77% of the total sample (response rate). Sociodemographic characteristics, violence and mental health outcomes are

presented in Table 1. The proportions of men and women were similarly distributed. The mean age of the total sample was 35.8 years (SD=18.8), and the average period of formal education was 8.8 (SD=4.7) years. Most people (57.2%) lived with a partner and the predominant races were Brown (44.3%) and White (40.1%).

Table 1. Characteristics of sociodemographic, violence and mental health variables.

Variable	Total Sample N=4607	% Weighted
**Sociodemographic**		
Sex		
Male	2,070	47.9
Female	2,537	52.1
Age		
14-18	1,229	11.9
19-30	952	26.0
31-59	1,779	47.3
60-99	647	14.8
Marital Status		
Single/separated/widowed	1,907	31.8
Living with partner	2,120	57.18
Race		
White	1,828	40.1
Black	570	12.6
Brown	2,074	44.3
Others	126	3.0
Brazilian regions		
North	417	7.4
Northeast	1,262	27.2
Midwest	317	7.2
Southeast	1,926	43.2
South	685	14.9
**Violence**		
Involvement in Fights		
No	4,442	97.4
Yes	160	2.6
Police Detention		
No	4,536	98.6
Yes	66	1.4
Domestic Violence		
No	1,926	91.6
Yes	191	8.4
**Mental Health**		
Social Support		
Low	1,357	28.9
High	3,250	71.1
Depression		
Yes	1,167	25.1
No	3,338	74.9
Alcohol dependence		
Yes	390	10.0
No	4,217	90.0

Domestic violence was the most prevalent category of aggressive behavior (8.4%) in the past twelve months, followed by involvement in fights (2.6%) and arrests by the police (1.4%). The total sample population showed high levels of social support (71%). Regarding mental health, depression was the most prevalent mental health condition (25%), and alcohol dependence were diagnosed in fewer than 10% of the responders.

More than 90% of the participants had a religious affiliation and 80.9% considered it "very important to their lives" (Table 2). In the bivariate analysis (Chi-square tests), having a religious affiliation was significantly associated with less violent outcomes, whereas the importance attributed to religion was only associated with less fights.

Table 2. Description of the religiosity on different outcomes of violence.

Variables	Total Sample N=4607	%*	Involvement in fights					Police detention					Domestic violence[1]				
			Yes		No			Yes		No			Yes		No		
			N	%*	N	%*	p-value ł	N	%*	N	%*	p-value ł	N	%*	N	%*	p-value ł
Religious Affiliation																	
Catholic	2,888	64	72	1.2	2,813	62.8	**0.001**	40	0.8	2,846	63.2	**0.001**	119	5.3	1,251	59.9	**0.001**
Protestant	1,108	23.5	41	0.7	1,065	22.8		8	0.1	1,099	23.4		40	1.6	482	22.3	
Others	153	3.1	10	0.2	143	8.7		2	0	150	3.1		1	0	64	0.3	
None	448	9.3	36	0.6	412	3		16	0.4	431	8.9		29	1.3	127	6.1	
Importance of Religion																	
Very important	3,369	80.9	80	1.4	3,285	79.5	**0.001**	42	1	3,324	80	0.826	127	6	1,503	77.2	0.183
Important	579	14.2	27	0.4	551	13.7		7	0.1	572	14		24	1.1	221	11.3	
Indifferent	175	4.1	11	0.3	164	3.8		1	0	173	4.1		9	0.4	66	3.2	
Few/not important	36	0.7	6	0	30	0.6		0	0	36	0.7		2	0.1	9	0.5	

Legend: *=weighted percentage. ł= p-value performed by Chi-square test (CI:95%). [1]=data from married people or living with partner.

The regression models between violence variables and religiosity are presented in Table 3. After all adjustment, religious affiliation showed an inverse association with fights (*OR*=0.60; 95%CI [0.37-0.98]) and police detention (*OR*=0.37; 95%CI [0.20-0.70]), but not with domestic violence (*OR*=0.57; 95% CI [0.30-1.08]). On the other hand, the importance of religion was also significantly associated with less involvement in fights (*OR*=0.60; 95% CI [0.36-0.99]).

Table 3. Associations between different outcomes of violence and religiosity.

	**Outcome**					
	Involvement in fights		*Police detention*		*Domestic violence*[1]	
	No	*Yes*	*No*	*Yes*	*No*	*Yes*
Religious Afiliation						
Crude	1.00	0.33 (0.20-0.55)*	1.00	0.26 (0.13-0.52)*	1.00	0.33 (0.21-0.67)*
OR (a)		0.46 (0.28-0.77)**		0.31 (0.15-0.63)**		0.48 (0.25-0.89)*
OR (b)		0.49 (0.30-0.79)**		0.30 (0.15-0.61)**		0.46 (0.25-0.88)*
OR (c)		0.49 (0.30-0.79)**		0.30 (0.15-0.61)**		0.47 (0.25-0.89)*
OR (d)		0.60 (0.37-0.98)*		0.37 (0.20-0.70)**		0.57 (0.30-1.08)
Importance of Religion						
Crude	1.00	0.34 (0.20-0.56)*	1.00	DN/A	1.00	DN/A
OR (a)		0.45 (0.26-0.74)**				
OR (b)		0.46 (0.28-0.75)**				
OR (c)		0.48 (0.29-0.78)**				
OR (d)		0.60 (0.36-0.99)*				

Legend: [1] Not adjusted for marital status. a) Adjusted for sociodemographic variables (age, gender, education, marital status, family income). b) Adjusted for sociodemographic and mental health variables (age, gender, education, marital status, family income, depression). c) Adjusted for sociodemographic and mental health variables and social support. c) Adjusted for sociodemographic and mental health variables (including social support) and alcohol dependency. *: p<0.05. **:p<0.01. DN/A: do not apply.

Table 4 presents age group models. Domestic violence could not be stratified in the age groups category as it was already stratified regarding marital status (only the participants who were married/living with a partner). Religious affiliation among adolescents (14-18) was inversely associated with violent behaviors in all analyses performed, losing significance when alcohol dependence was added. In the age group ranging from 19 to 30 years old, religious affiliation was inversely associated with police detention even after all adjustments. Finally, in

the age group between 31 to 59 years old, only the importance of religion was associated with less involvement in fights, finding not maintained after adjustments.

Table 4. Associations between violence outcomes and religiosity stratified by age group, Brazil, 2012.

	Outcome			
	Involvement in fights		*Police Detention*	
	No	Yes	No	Yes
Religious Affiliation				
14-18 (a)	1.00	0.42 (0.20-0.86)*	1.00	0.24 (0.08-0.69)**
14-18 (b)		0.61 (0.30-1.23)		0.42 (0.12-1.53)
19-30 (a)		0.57 (0.21-1.50)		0.20 (0.06-0.63)*
19-30 (b)		0.65 (0.24-1.72)		0.22 (0.07-0.65)**
31-59 (a)		0.52 (0.17-1.62)		1.40 (0.18-10.58)
31-59 (b)		0.66 (0.19-2.31)		2.00 (0.28-14.43)
Importance of Religion				
14-18 (a)	1.00	0.48 (0.27-0.85)*		DN/A
14-18 (b)		0.59 (0.33-1.04)		DN/A
19-30 (a)		0.57 (0.24-1.36)		DN/A
19-30 (b)		0.73 (0.29-1.83)		DN/A
31-59 (a)		0.39 (0.18-0.86)*		DN/A
31-59 (b)		0.49 (0.22-1.13)		DN/A

Legend: a) Adjusted for sociodemographic variables (age, gender, education, marital status, depression, support social). b) Adjusted for (a) and alcohol dependence. *: p<0.05. **: p<0.01. DN/A: do not apply.

In the mediation analyses, the incorporation of social support variable in the regression model produced no significant changes in the effect of religiosity; social support did not have a mediating role in this model. On the other hand, stratified analyses pointed that alcohol dependence could have a mediation role between religiosity and violence outcomes. Therefore, to explore the mediation effect, we used chi-square (95%CI) test to determine the association between alcohol dependence and religious affiliation (χ^2= 80.57; p=0.001), importance of

religion (χ^2= 67.26; p=0.001), involvement in fights (χ^2= 135.11; p=0.001), police detention (χ^2= 53.95; p=0.001) and domestic violence (χ^2= 107.22; p=0.001). This variable met all criteria for inclusion as a possible mediator.

Figure 1 shows the analyses of the mediation effect tested for religious affiliation with involvement in fights, police detention and domestic violence, and the mediation effect of the importance of religion with involvement in fights. Religious affiliation was totally mediated by alcohol dependence in relation to its effect on domestic violence. However religious affiliation was partially mediate by alcohol dependence in involvement in fights and police detention and the importance of religion was partially mediated by alcohol dependence in involvement in fights.

Figure 1. Alcohol dependence as a mediator: indirect effect of religiosity on violence.

Discussion

In this nationwide representative population based-study of 4,607 Brazilian individuals from the general population, religiosity was inversely associated with different violent outcomes, in that individuals having a religious affiliation were less involved in fights and detention; and those who considered religion important in their lives were also less involved in fights. This relationship seems to be mediated by alcohol dependence and is potentially different among age groups. These results could add to the current scientific literature and will be explained further below.

Our findings corroborate with previous studies, showing that organizational religiosity (i.e. religious affiliations and religious attendance) may have a larger effect on violence than intrinsic or self-reported religiosity (i. e. the importance of religion in life) [8]. The first explanation for this effect is the role that religion plays in social control. A formal religion usually engages individuals in volunteering and philanthropic behaviors, as well as provides psychosocial adjustment and social support, most commonly presenting non-violent behaviors as its doctrine or thinking's [29,30], which could minimize violent behaviors. Providing support to this hypothesis, Resnick et al. [14] investigated fights, threats, stabbings and shootings in a representative sample of American adolescents and concluded that youths who attended religious services and had greater value for their religion were less involved in violence. In the same line, a previous study found that not having a religion was a risk factor for intimate partner violence in Brazil [31] and others found that carrying a weapon was inhibited among individuals with high levels of R/S [19,32]. Religious social control theory seems to support those findings independently of the violence behavior perpetrated.

The second explanation is the human development theory, in which the essence of religion, meaning and purpose of life, becomes more prominent as age advances to a more reflective stage of life [33,34]. The impact of the importance of religion was more noted in adults than in adolescents. These results are supported by previous studies which found less engagement in gangs and fights for inmates above 24 years of age who had private religiosity, but not for those who attended religious services [19]. Another study noted that a decrease in theft was predominantly the result of religious attendance in adolescents and the result of private religiosity in young adults [35]. The attribution of importance to religion is considered a

private religiosity, a concept independent of a formal religion, which can raise consciousness to prevent destructive social behavior [36].

In addition, R/S is a multidimensional concept consisting of different pathways to influence human behavior. Concerning the role of mediators in this relationship, social development factors, such as antisocial bonding and beliefs, were entirely responsible for mediating different measures of R/S and violence on a sample of adolescents [32]. To our knowledge, however, no study has explored social support as an indirect effect of religiosity on violence. It is possible that encouraging positive social behavior may be a more distinguished pathway in which religiosity may operate.

Alcohol dependence, on the other hand, had an important role as mediator, attenuating religiosity and maintaining the effect in different age groups. In a study using the first database of BNADS demonstrated that individuals with high levels of R/S had more supporting opinions concerning alcohol control policies, such as limiting hours of sales and advertisements, raising the legal drinking age and increasing taxes [17]. It is possible that the prohibitive role of religion on alcohol as a licit drug is more evident than that in illicit drugs, which can be more of a secular institutional problem to be controlled.

The observed association between religious affiliation on domestic violence in entirely mediated by the effect of alcohol abuse/dependence. These findings corroborated by previous findings, which showed that heavy episodes of drinking mediated the protective effect of church attendance on sexual aggression in a follow-up period of 2 years in university students [18] and by a study that showed that discussing spiritual/moral values to partners, victims and perpetrators of domestic violence, resulted in a 71% reduction in the cases of violence [37]

It is not unusual to find modest results of R/S mediation analysis [38,39], probably because of the complexity of this multidimensional concept. We suggest further investigation of other mental health disorders as possible mediators of R/S and violence, such as antisocial and borderline personality disorders, which can be associated with more aggressive behaviors [40]. Furthermore, interventional methodological designs to reduce aggressive and criminal behavior have been showing satisfactory results [37,41] and should be considered when exploring possible mechanisms of action of the R/S dimension.

Limitations

The present study has some limitations that should be considered. First, although we assessed two important and previously studied variables [7], it is notable that the R/S dimension should be treated as a multidimensional concept and assessed in different measures. The use of more detail and validated questionnaires to assess R/S instead of the uso of single questions could have found different results. Second, our study design was cross-sectional and, therefore, no inferences of causal-effect relationship could be made. Third, although this is a nation-wide representative survey, it reflects the Brazilian scenario. Some authors suggest that cultural aspects may interfere with the religiosity and violence relationship [29,37]. Although the most recent meta-analysis did not find differences between studies performed in the United States of America and studies in other countries [8], it is worth mentioning that Brazil is a highly religious country [42]. Thus, cultural influences should be considered when interpreting the results of the present study.

Conclusions

Religiosity seems to be an important factor associated with lower levels of violence in this nationwide representative survey, across all age groups investigated. Alcohol dependence seems to mediate this relationship. Health professionals should be aware of these findings in their clinical practice. The role of the religious/spiritual dimension in violence needs to be further investigated in order to justify its inclusion of complementary medical approaches.

Funding

This work received financial support from CNPq-Brazilian Council for Scientific and Technological Development (GL, HV and RL) and from CAPES-Brazilian Federal Government Agency (JPBG).

Acknowledgments

We would like to thank Cleusa Ferri for her comments and advice on this work.

References

1. Butchart A, Mikton C, Dahlberg LL, Krug EG. Global status report on violence prevention 2014. Inj Prev [Internet]. 2015;21(3):213. Available from: https://www.ncbi.nlm.nih.gov/pubmed/25995201
2. Collaborators GB of DS 2013. Global, regional, and national incidence, prevalence, and years lived with disability for 301 acute and chronic diseases and injuries in 188 countries, 1990-2013: a systematic analysis for the Global Burden of Disease Study 2013. Lancet [Internet]. 2015;386(9995):743–800. Available from: https://www.ncbi.nlm.nih.gov/pubmed/26063472
3. Hughes K, Bellis MA, Hardcastle KA, Butchart A, Dahlberg LL, Mercy JA, et al. Global development and diffusion of outcome evaluation research for interpersonal and self-directed violence prevention from 2007 to 2013: A systematic review. Aggress Violent Behav. 2014;19(6):655–62.
4. Wenzel T, Kienzler H, Wollmann A. Facing Violence - A Global Challenge. Psychiatr Clin North Am. 2015;38(3):529-+.
5. Koenig H, King D, Carson V. Handbook of religion and health. 2012. 1169 p.
6. Baier CJ, Wright BRE. “If you love me, keep my commandments”: A meta-analysis of the effect of religion on crime. J Res Crime Delinq. 2001;38(1):3–21.
7. Johnson BR, LI S De, Larson DB, McCullough M. A Systematic Review of the Religiosity and Delinquency Literature: A Research Note. J Contemp Crim Justice [Internet]. 2000;16(1):32–52. Available from: https://doi.org/10.1177/1043986200016001003
8. Kelly PE, Polanin JR, Jang SJ, Johnson BR. Religion, Delinquency, and Drug Use: A Meta-Analysis. Crim Justice Rev [Internet]. 2015;40(4):505–23. Available from: https://doi.org/10.1177/0734016815605151
9. Hirschi T, Stark R. Hellfire and delinquency. Soc Probl. 1969;17(2):202–13.

10. Ellis L. Religiosity and criminality – Evidence and explanations of complex relationships. Sociol Perspect [Internet]. 1985;28(4):501–20. Available from: %3CGo
11. Cochran JK, Wood PB, Arneklev BJ. Is the religiosity-delinquency relationship spurious – a test of arousal and social-control theories. J Res Crime Delinq [Internet]. 1994;31(1):92–123.
12. Pitel L, Madarasova Geckova A, Kolarcik P, Halama P, Reijneveld SA, van Dijk JP. Gender differences in the relationship between religiosity and health-related behaviour among adolescents. J Epidemiol Community Heal [Internet]. 2012;66(12):1122–8. Available from: https://www.ncbi.nlm.nih.gov/pubmed/22569749
13. Salas-Wright CP, Vaughn MG, Schwartz SJ, Cordova D. An "immigrant paradox''' for adolescent externalizing behavior? Evidence from a national sample." Soc Psychiatry Psychiatr Epidemiol [Internet]. 2016;51(1):27–37. Available from: %3CGo
14. Resnick MD, Ireland M, Borowsky I. Youth violence perpetration: what protects? What predicts? Findings from the National Longitudinal Study of Adolescent Health. J Adolesc Heal [Internet]. 2004;35(5):424.e1-10. Available from: https://www.ncbi.nlm.nih.gov/pubmed/15488438
15. Baier D. The influence of religiosity on violent behavior of adolescents: a comparison of Christian and Muslim religiosity. J Interpers Violence [Internet]. 2014;29(1):102–27. Available from: https://www.ncbi.nlm.nih.gov/pubmed/24114763
16. Nonnemaker JM, McNeely CA, Blum RW. Public and private domains of religiosity and adolescent health risk behaviors: evidence from the National Longitudinal Study of Adolescent Health. Soc Sci Med [Internet]. 2003;57(11):2049–54. Available from: %3CGo
17. Lucchetti G, Koenig HG, Pinsky I, Laranjeira R, Vallada H. Religious beliefs and alcohol control policies: a Brazilian nationwide study. Rev Bras Psiquiatr [Internet]. 2014;36(1):4–10. Available from: https://www.ncbi.nlm.nih.gov/pubmed/24346358
18. Kingree JB, Thompson M, Ruetz E. Heavy Episodic Drinking and Sexual Aggression Among Male

College Students: The Protective Influence of Church Attendance. J Interpers Violence [Internet]. 2015; Available from: https://www.ncbi.nlm.nih.gov/pubmed/26002879

19. Benda BB, Toombs NJ. Religiosity and violence: Are they related after considering the strongest predictors? J Crim Justice [Internet]. 2000;28(6):483–96. Available from: http://www.sciencedirect.com/science/article/pii/S0047235200000647

20. Ally EZ, Laranjeira R, Viana MC, Pinsky I, Caetano R, Mitsuhiro S, et al. Intimate partner violence trends in Brazil: data from two waves of the Brazilian National Alcohol and Drugs Survey. Rev Bras Psiquiatr [Internet]. 2016;38(2):98–105. Available from: https://www.ncbi.nlm.nih.gov/pubmed/27304756

21. Caetano R, Vaeth PA, Ramisetty-Mikler S, Rodriguez LA. The Hispanic Americans baseline alcohol survey: alcoholic beverage preference across Hispanic national groups. Alcohol Clin Exp Res [Internet]. 2009;33(1):150–9. Available from: https://www.ncbi.nlm.nih.gov/pubmed/18976346

22. Batistoni SST, Neri AL, Cupertino A. Validity of the Center for Epidemiological Studies Depression Scale among Brazilian elderly. Rev Saude Publica. 2007;41(4):598–605.

23. Brugha TS, Morgan Z, Bebbington P, Jenkins R, Lewis G, Farrell M, et al. Social support networks and type of neurotic symptom among adults in British households. Psychol Med [Internet]. 2003;33(2):307–18. Available from: https://www.ncbi.nlm.nih.gov/pubmed/12622309

24. Quintana MI, Andreoli SB, Jorge MR, Gastal FL, Miranda CT. The reliability of the Brazilian version of the Composite International Diagnostic Interview (CIDI 2.1). Braz J Med Biol Res [Internet]. 2004;37(11):1739–45. Available from: https://www.ncbi.nlm.nih.gov/pubmed/15517091

25. Rucker DD, Preacher KJ, Tormala ZL, Petty RE. Mediation Analysis in Social Psychology: Current Practices and New Recommendations. Soc Personal Psychol Compass [Internet]. 2011 Jun 1;5(6):359–71. Available from: https://doi.org/10.1111/j.1751-9004.2011.00355.x

26. Baron RM, Kenny DA. The moderator-mediator variable distinction in social psychological

research: conceptual, strategic, and statistical considerations. J Pers Soc Psychol. 1986;51(6):1173–82.

27. MacKinnon DP, Lockwood CM, Hoffman JM, West SG, Sheets V. A comparison of methods to test mediation and other intervening variable effects. Psychol Methods [Internet]. 2002;7(1):83–104. Available from: https://www.ncbi.nlm.nih.gov/pubmed/11928892

28. Preacher KJ, Kelley K. Effect size measures for mediation models: quantitative strategies for communicating indirect effects. Psychol Methods. 2011 Jun;16(2):93–115.

29. Cheung CK, Yeung JWK. Meta-analysis of relationships between religiosity and constructive and destructive behaviors among adolescents. Child Youth Serv Rev. 2011;33(2):376–85.

30. Burkett SR, Ward DA. A note on perceptual deterrence, religiously based moral condemnation, and social-control. Criminology. 1993;31(1):119–34.

31. Zaleski M, Pinsky I, Laranjeira R, Ramisetty-Mikler S, Caetano R. Intimate partner violence and contribution of drinking and sociodemographics: the Brazilian National Alcohol Survey. J Interpers Violence [Internet]. 2010;25(4):648–65. Available from: https://www.ncbi.nlm.nih.gov/pubmed/19491308

32. Salas-Wright CP, Olate R, Vaughn MG, Tran T V. Direct and mediated associations between religious coping, spirituality, and youth violence in El Salvador. Rev Panam Salud Publica-Pan Am J Public Heal [Internet]. 2013;34(3):183–9. Available from: %3CGo

33. Gormly A V. Lifespan: Human Development. Holt Rinehart & Winston.; 1993.

34. Kohlberg L. Essays on moral development: Vol. 2. The psychology of moral development: Moral stages, their nature and validity. <p class="MsoNormal" style="margin-bottom:0cm;margin-bottom:.0001pt;line-height: normal;mso-layout-grid-align:none;text-autospace:none"> San Francisco: Harper & Row.; 1984.

35. Salas-Wright CP, Vaughn MG, Maynard BR. Buffering Effects of Religiosity on Crime Testing the

Invariance Hypothesis Across Gender and Developmental Period. Crim Justice Behav. 2014;41(6):673–91.

36. Fernander A, Wilson JF, Staton M, Leukefeld C. Exploring the type-of-crime hypothesis, religiosity, and spirituality in an adult male prison population. Int J Offender Ther Comp Criminol [Internet]. 2005;49(6):682–95. Available from: https://www.ncbi.nlm.nih.gov/pubmed/16249398

37. Puchala C, Paul S, Kennedy C, Mehl-Madrona L. Using traditional spirituality to reduce domestic violence within aboriginal communities. J Altern Complement Med [Internet]. 2010;16(1):89–96. Available from: https://www.ncbi.nlm.nih.gov/pubmed/20055557

38. Edlund MJ, Harris KM, Koenig HG, Han XT, Sullivan G, Mattox R, et al. Religiosity and decreased risk of substance use disorders: is the effect mediated by social support or mental health status? Soc Psychiatry Psychiatr Epidemiol. 2010;45(8):827–36.

39. VanderWeele TJ, Li SS, Tsai AC, Kawachi I. Association Between Religious Service Attendance and Lower Suicide Rates Among US Women. Jama Psychiatry [Internet]. 2016;73(8):845–51.

40. Shorey RC, Elmquist J, Anderson S, Stuart GL. The Relationship between Antisocial and Borderline Features and Aggression in Young Adult Men in Treatment for Substance Use Disorders. J Psychoact Drugs [Internet]. 2016;48(2):115–23. Available from: https://www.ncbi.nlm.nih.gov/pubmed/26941068

41. Duwe G, Hallett M, Hays J, Jang SJ, Johnson BR. Bible college participation and prison misconduct: a preliminary analysis. 2015;54:371–90.

42. Peres MFP, de Oliveira AB, Leão FC, Vallada H, Moreira-Almeida A, Lucchetti G. Religious landscape in Brazil: Comparing different representative nationwide approaches to obtain sensitive information in healthcare research. SSM - Popul Heal [Internet]. 2018 Sep 7;6:85–90. Available from: https://www.ncbi.nlm.nih.gov/pubmed/30246139

Figure Click here to download Figure Figure 1. general mediation.tif

Figure 1.Mediation role alcohol dependence on religiosity and violence outcomes

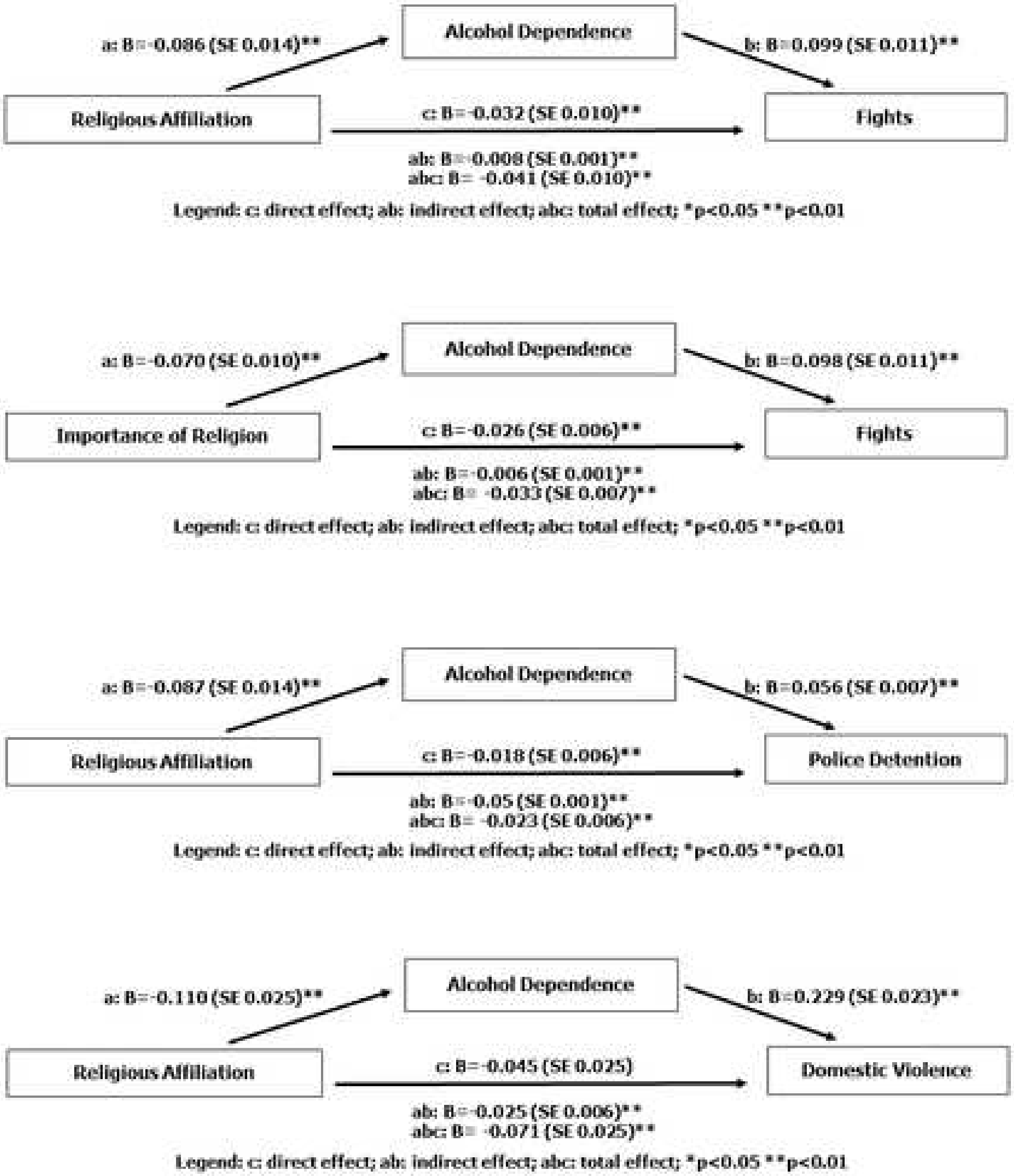

Supplement: Attachment — Submitted filename: PONE-D-20-08721_reviewer_LM_04_08_2020.pdf [file pone.0238020.s002.pdf]
